# Ontogenetic variations in the venom proteome of the Amazonian snake *Bothrops atrox*

**DOI:** 10.1186/1477-5956-4-11

**Published:** 2006-05-11

**Authors:** Rafael AP Guércio, Anna Shevchenko, Andrej Shevchenko, Jorge L López-Lozano, Jaime Paba, Marcelo V Sousa, Carlos AO Ricart

**Affiliations:** 1 Brazilian Center for Protein Research, Department of Cell Biology, University of Brasilia, Brasília, 70910-900- DF, Brazil; 2 Max Planck Institute of Molecular Cell Biology and Genetics, Pfotenhauerstrasse 108, 01307 Dresden, Germany; 3 Gerência de Animais Peçonhentos – Fundação de Medicina Tropical do Amazonas, Av. Pedro Teixeira 25, 69040-000 Manaus, AM, Brazil

## Abstract

**Background:**

*Bothrops atrox *is responsible for the majority of snakebite accidents in the Brazilian Amazon region. Previous studies have demonstrated that the biological and pharmacological activities of *B. atrox *venom alter with the age of the animal. Here, we present a comparative proteome analysis of *B. atrox *venom collected from specimens of three different stages of maturation: juveniles, sub-adults and adults.

**Results:**

Optimized conditions for two-dimensional gel electrophoresis (2-DE) of pooled venom samples were achieved using immobilized pH gradient (IPG) gels of non-linear 3–10 pH range during the isoelectric focusing step and 10–20% gradient polyacrylamide gels in the second dimension. Software-assisted analysis of the 2-DE gels images demonstrated differences in the number and intensity of spots in juvenile, sub-adult and adult venoms. Although peptide mass fingerprinting (PMF) failed to identify even a minor fraction of spots, it allowed us to group spots that displayed similar peptide maps. The spots were subjected to a combination of tandem mass spectrometry and Mascot and MS BLAST database searches that identified several classes of proteins, including metalloproteinases, serine proteinases, lectins, phospholipases A_2_, L-amino oxidases, nerve growth factors, vascular endothelial growth factors and cysteine-rich secretory proteins.

**Conclusion:**

The analysis of *B. atrox *samples from specimens of different ages by 2-DE and mass spectrometry suggested that venom proteome alters upon ontogenetic development. We identified stage specific and differentially expressed polypeptides that may be responsible for the activities of the venom in each developmental stage. The results provide insight into the molecular basis of the relation between symptomatology of snakebite accidents in humans and the venom composition. Our findings underscore the importance of the use of venoms from individual specimen at various stages of maturation for the production of antivenoms.

## Background

The genus *Bothrops *(family *Viperidae*) comprises several species of pit vipers inhabiting the American continent from Mexico to Argentina [1]. *Bothrops atrox *species are responsible for the majority of snakebite accidents in the Brazilian Amazon region [2]. In humans, *Bothrops atrox *envenomation causes local effects such as swelling, local hemorrhage and necrosis besides systemic effects, including alterations in blood coagulation and various types of bleeding distant from the bite site [3]. Perturbed blood hemostasis and thrombosis are largely caused by proteinases, especially metallo- and serine- proteinases that are the major components of *Bothrops *snake venoms [4].

Among other factors, the composition of snake venoms is affected by the age of the animals. A comparative study of the proteinase activity and protein profiles of venoms from juvenile, sub-adult and adult *Bothrops atrox *specimens captured in the Brazilian Amazon rain forest was previously reported [2]. López-Lozano et al demonstrated that venoms from juveniles and sub-adults displayed higher human plasma clotting activity compared to adult venoms. In addition, SDS-PAGE and HPLC venom protein profiles varied among the three developmental stages analyzed. Two proteins of 23 kDa and 50 kDa, respectively, that were present in higher amounts in adult venoms, were identified as metalloproteinases.

An independent study of *B. atrox *specimens from the Colombian Amazon rain forest showed that venoms of newborn and juvenile specimen caused higher lethality and possessed higher hemorrhagic and coagulant activities, than adult venoms. The differences in activity were attributed to the increased amount of high molecular mass proteins, probably also metalloproteinases [5].

Taken together, these and other published evidence indicated that changes in the venom proteome during ontogenetic development can influence its biological activity. Here we report a comparative proteome analysis of *B. atrox *venoms from juvenile, sub-adult and adult specimens that identified proteins whose differential expression during ontogenetic development may be correlated to the previously reported properties of the venom [2,5].

## Results

In order to optimize 2-DE separation of *B. atrox *venom proteins, linear and non-linear 3–10 pH gradients were tested in the isoelectric focusing (IEF) step. The non-linear gradient, developed to improve resolution of acidic proteins, provided better resolution of spots than linear pH gradient since many spots consisted of polypeptides displaying isoelectric points (p*I*) between 4 and 7 (data not shown). Two types of electrophoresis equipment – Multiphor II and IPGphor from GE Healthcare- were tested for the IEF step and both provided similar 2-DE maps (data not shown). On the other hand, for the second dimension, gradient gels (10–20% T) provided better 2-DE maps than 12% T gels, especially for proteins with molecular masses around 14 kDa (data not shown).

**Figure 1:**
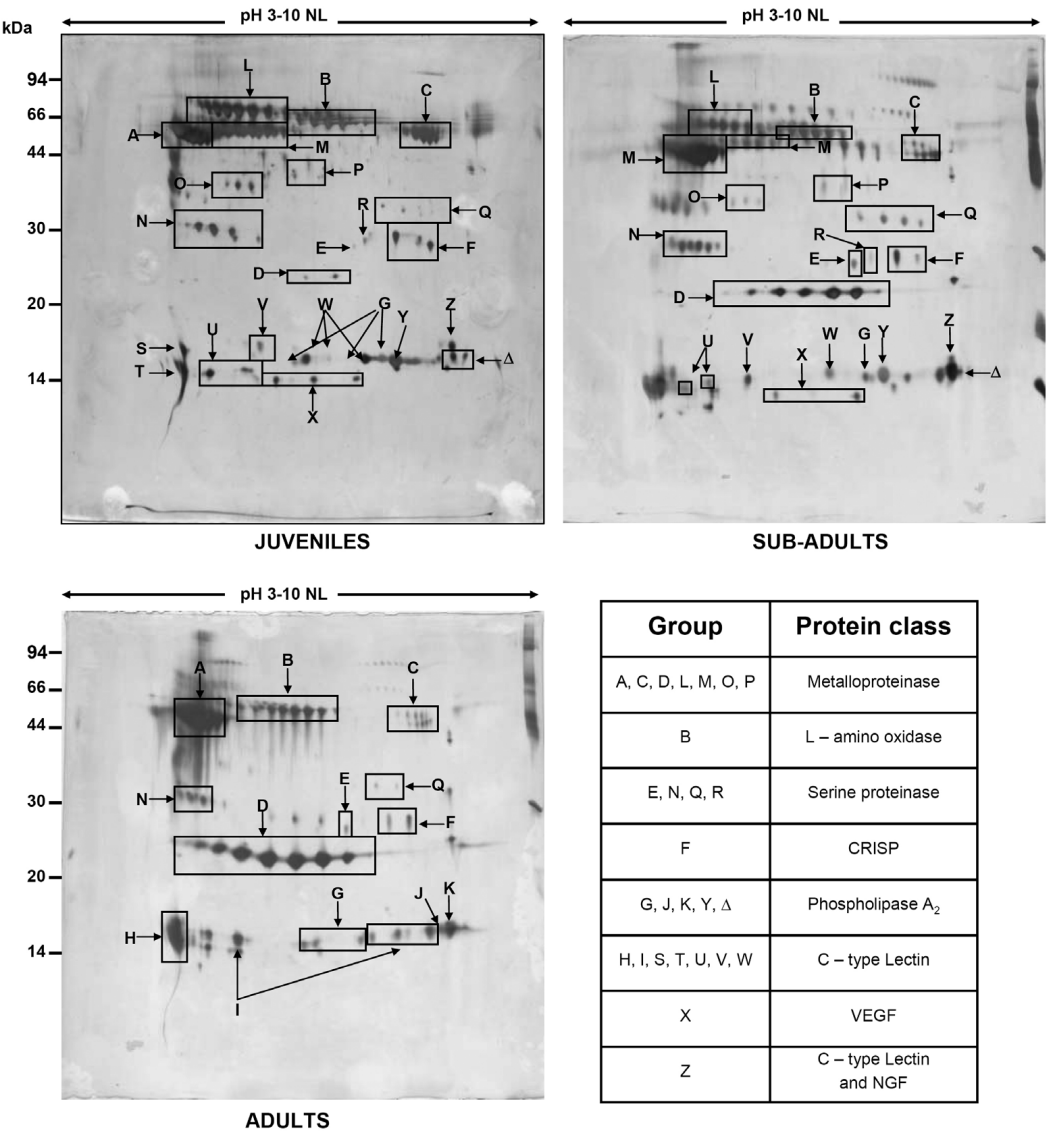
Proteome maps of *B. atrox *venom from juveniles, sub-adults and adults. Spots displaying similar peptide mass fingerprints were grouped as explained in Results section. A summary of the protein classes identified in each group is also shown.

Patterns of protein spots visualized by silver staining were different between pooled venom samples from juvenile, sub-adult and adult *B. atrox *(Fig. 1). Their computer-assisted image analyses detected 110 spots in the gels from juveniles, 101 in sub-adults and 86 in adult venoms. Among the detected spots, 44 were found specifically in juveniles, 22 in sub-adults and 22 in adults. Image analysis also pinpointed substantial differences in the relative abundance of several spots matched at all three images (Table 1).

**Table 1:** Protein groups identified in *B. atrox *venom proteome. Groups from A to Δ comprise spots that displayed similar peptide mass fingerprints shown in Fig 3. p*I *range, molecular mass (MM) and relative expression were determined by computational analyses of the 2-DE gels. X: unknown amino acid; B: cleavage site; J: juveniles; S: sub-adults, A: adults.

Group	Protein Class	Best Match sptrembl # Organism	Peptide sequences	p*I *range	MM (kDa)	Relative expression
A	Metalloproteinase	Bothropasin O93523 *Bothrops jararaca*	BITVKPDVDYTLNSFAEWR BASMSECDPAEHCTGQSSECPADVFHK BMYELANIVNEIFR BKIPCAPEDVK BKTDLLTR BGMVLPGTK BXXFQDVYEAEDSCFK QYNPFR YLEFLLVVDQLLNK BYNPFR	5.0	52	J = S = A
B	L-amino oxidase	L-amino oxidase I Q6TGQ9 *Bothrops jararacussu*	BXXGQLYEESLQK BLFLTMNK BXXTVTYQAVMK BIKFEPPLPPKK BRFDEIVGGMDK BHDDIFAYEK BXXWYANLGPMRLPEK BFWEDDGIHGGK BETDYEEFLEIAK	5.9 –6.2	55 – 61	J = S = A
C	Metalloproteinase	Bothropasin O93523 *Bothrops jararaca*	BXXVEVGEECDCGSPR BXXGTECQAA DMADLCTGR BLYCVDSAVNGR BLYCVDSSPANK BGMVLPGTK BKIPCAPEDVK BYNPFR BXXFQDVYEAEDSCFK	7.1–7.5	40 – 51	J>S>A
D	Metalloproteinase	Metalloproteinase Q8QG89 *Bothrops insularis*	BTLDSFGEWR-BTLDSFEGWR BTLDSFWWR BTLDSFWEGR BTLDSFWGER BYVDLFLVVDHGVLDNK BYVDLFLVVDHGMFMK- BYVDLFLVVDHGXXXK BDLINVQQDSR BENPQCILNKR	5.3 – 6.4	23–24	J<S<A
E	Serine proteinase	Bilineobin Q9PSN3 *Agkistrodon bilineatus*	BVVGGDECNINEHR BSLPSSPPSVWSASK BSLPSSPPSVXXSASK BDIMLIR BVMGWGSLSSPK BFLCNPR BFLCGGPR BIFLTCTK	6.3 –6.4	28 – 29	J = S = A
F	CRISP	Ablomin Q8JI40 *Agkistrodon halys blomhoffi*	BSVNPTASNMLK BMEWYPEAAANAER BKPEIQNEIVDLHNSLR BSGPPCGDCPSACDDGLCTNPCTK AGCAAAYCPSSSYK BXXDFDSESPR	7.1 –8.1	28 – 29	J>S = A
G	Phospholipase A_2_	Phospholipase A_2 _ Q7ZTA7 *Crotalus viridis*	BVAVLCFR FVHDCCYGK BMDLYTLHR-BMDLYTYDK	8.1	16	J = S = A
H	C-type lectin	Botrocetin β chain P22030 *Bothrops jararaca* Platelet glycoprotein IB-binding protein Q9PSM5 *Bothrops jararaca* Phospholipase A_2 _ Q8QG87 *Bothrops insularis*	BDCPSGWSSYEGSCYK FVVTEYECVASK BXXSFEWSDGTDLSYK BXXTQLAYVVCK -BLAYVVCEAQR -BLAYVVCK BTCLGLEEDSGFR BSLTSPMLK DNQWLSFPCTR BXXNAWLGLWEGK BXXSFEWSDGTDLTYK BXXWADAESLCALQR BTCLGLEEDSGFRK BFCTLQQR BLLQLLQR BEEADFVR BNFVCEFQA BWWIIPCTR BVAAICFR BVAATCFR BYWLYGAK	4.8	17	J = S<A
I	C-type lectin	Botrocetin α chain P22029 *Bothrops jararaca*	BDCPSGWSSYEGNCYK BLYSGEADFVGDLVTK BWSDGSSVSYENVVER BMNWADAER BKCFALEK BGGHLVSIK BCFALEK BNPFVCK BFFQQK	5.3	15	J = S<A
J	Phospholipase A_2_	Myotoxin I P20474 *Bothrops asper*	BAAAVCFR BSLIEFAK BMILEETK BKSGVIICGEGTPCEK BXXAYPDLFCK BLYSGEADFVVK BVAVLCFR BAAGLCGFR BVTGVPTYK BSGVIICGE	7.8	17	J = S<A
K	Phospholipase A_2_	Myotoxin III Q9PVE3 *Bothrops asper*	BXXVCDENNPCLK BXXVCDENNPPGR-VCDENNPCLK BYFAYPDLFCK VTSYQY BMILQETGKNPVTSYGAYGCNCGVLGR BELCECDKAVAICLR BAVAICLR BYSYSWK	7.8	17	J = S<A
L	Metalloproteinase	Berythractivase Q8UVG0 *Bothrops erythromelas*	BLTPGSQCADGLCCDQCR BKYVEFVVVLDHGMYK BVPLTGLELWSDR BLYCFLYSSK VVFEPFK-VVQHQVR BVPLTXVLDHRFK KIPCAPEDVK	5.0 –5.9	61 – 66	J>S>A
M	Metalloproteinase	BOJUMET II Q7T1T5 *Bothrops jararacusu*	BETVLLNR BYLIDNRPPCILNIPLR BFALVGLEMWSNR BFALVGLDMGWSNR BSSDLGMVDLASK VQGPLGNTLTCM PTDTDFDGTLLGLAWR TDTDFDGTLLGLAWR GQSADCPTDDLQR	5.4 –5.7	51–53	J>S>A
N	Serine proteinase	Serine proteinase Q7T229 *Bothrops jararacusu*	BLVGGDECNINEHR BIMGWGTLSPTK BFLCNPR BVSDYTEWLK	5.0 –5.3	29 – 30	J>S = A
O	Metalloproteinase	Metalloproteinase Q8AWX7 *Agkistrodon halys palas*	BYIELVIVADHR BSVGIVRDYR BSVANDDEVIRYPK	5.2 –5.4	36 – 37	J>S>A
P	Metalloproteinase	Factor X activator heavy chain Q7T046 *Vipera lebetina*	BLYETVNALNVLCR BYSVGLVQDYR BYIELVIVADHR BLNLNPDEQR	5.8 –6.2	38 – 40	J>S>A
Q	Serine proteinase	Serine proteinase Q8QG86 *Bothrops insularis*	BFLAFLYPGR BIYLGIHAR BDIMLIR BLHEPALYTK BLQGLVSDHR BSVANDDP-BSVANDDEE	6.3 –7.4	32 – 34	J = S>A
R	Serine proteinase	Catroxase II Q8QHK2 Crotalus atrox	BLDDVLDQDLG BTLCAGILEGGK BAAYPELPATSR BAAYPERFTSR BLPSNPPW-BLPSNPPWHR BLPSNPPXXXR	6.5	29	J = S>A
S	C-type lectin	Alboaggregin A subunit 2 P81112 *Trimeresurus albolabris*	WADAER SYENWTEAELK BTCLGLEEDSGFR BTCLALEEDSGFR FCAGYLENK BTPLNLNCR BXXLNLNCR BXXTADAER	4.8	17	J>S = A
T	C-type lectin	Mucrocetin Q6TPG9 *Trimeresurus mucrosquamatus*	BDCPSGWSSYEGSCYK BFCTQQQTNHLVSFQSR BXXCQFVVTEYPSFQSK BXXSFEWSDGTDLSYK BXXTQLAYVVCK BTTENQWWSR BXXLNLNCR BLFLQQNK BLTSPLLR BMNWEDAEK	4.8	15	J = S>A
U	C-type lectin	Alboagregrina A P81114 *Trimeresurus albolabris*	BDCPSDWSSYEGHCYR BVFNEPQNWADAEK BQSSEEADFVLK BSSEEADFVLK BXXLDQLLK BLFLQQNK	5.1–5.4	14	J = S>A
V	C-type lectin	Bothrocetin α chain P22029 *Bothrops jararaca*	BCFVLER BXXSDGSCVCYENLVER BEGFLTWR BSPMSPDTEEGK BXXCYENLVER	5.1–5.4	14	J = S>A
X	VEGF	Vascular Endothelial growth factor Q90X24 *Bothrops insularis*	CCTDESLECTATGK BLEVMKFTEHTNCECR BETLVSLLEEHPDE PSCVTALR BLFRAVLFNALR	5.6 –6.3	13	J>S>A
W	C-type lectin	Botrocetin α chain P22029 *Bothrops jararaca*	BDCPSGWSSYEGHCYR BXXWSDGSSVSYENLVER VSFQSDWTDFVVK BLVSFQSDWTDFVVK BLWADAER BNPFVCK BFCSEQAK BNIQSSDLYAWIGLR	5.9 –6.4	15	J = S>A
Y	Phospholipase A_2_	Phospholipase A_2 _ Q8QG87 *Bothrops insularis*	BYFSYGCYCGLGGLGQPR-GSYGCYCLGGL BXXFVHDCCYGK BXXXFVHDCCYGK BXXKDTYNLQYWLYQK-BXXKDTYNLKYWLYAGK BLTYNLQYWLYQK-BLTYNLKYWLYAAGK BYGEGLYQK-BYGEGLYAGK BVVTTCFR BVAVLCTR BQLCECDFVA BXXCECDFV BXXLWQFGT	6.7	15	J = S>A
Z	C-type lectin e NGF	Platelet glycoprotein IB-binding protein α chain Q9PSM6 *Bothrops jararaca* Nerve growth factor Q9DEZ9 *Crotalus durissus terrificus*	BQYFFETK BALTMEGNQASWR BIDTACVCVISR BXXALGQK BFIRPR BNPFVCK BFFQQK BDTPFECPSDWSTHR BXXSDGSCVCYENLVR	7.8	17	J = S>A
Δ	Phospholipase A_2_	Myotoxin III Q9PVE3 *Bothrops asper*	BSYAAYGCNCGVLGR BMLLLETGKLPAK NLWQLGK VAVLCFR BAVAICLR BYSYSWK BYNYLKPFCK BTIVCGENNSCLK	6.4 –8.1	16	J = S>A

The identification of proteins was initially attempted by N-terminal sequencing of the proteins blotted to a PVDF membrane using Edman degradation. Because of the relatively low sensitivity of Edman degradation reliable peptide sequences were retrieved only from the most abundant spots. For instance, the group of seven spots of 23 kDa, more abundant in adults (group D, Fig. 1), presented the same N-terminal sequence (TPEQQRYVELLXVVD), where X stands for an undetermined amino acid. This sequence was 73 % identical to a fragment of the 23 kDa metalloproteinase bothrolysin [sptrembl: P20416] (1 TPEHQRYIELFLVVD 15) and to an internal sequence of the 50 kDa metalloproteinase bothrostatin [sptrembl: Q98SP2] (188 TPEHQRYIELFLVVD 202), both from *B. jararaca*. However, Edman sequencing failed to identify a 52 kDa polypeptide that was detected as the most abundant spot in the 2-DE map of adult venom (group A, Fig. 1).

In-gel digests of non-identified spots were further analyzed by peptide mass fingerprinting (PMF). Only a few full length sequences of *B. atrox *proteins are currently available in a database, and therefore it is not surprising that PMF searches did not provide significant scores for any of the 2-DE spots present in the gels. However, this approach allowed us to group spots whose peptide mass fingerprints were similar. Based on the PMF data, all spots were arranged in 27 groups. The 2-DE maps of juveniles, sub-adults and adults showing the groups containing spots with similar PMF spectra are shown in Fig. 1. All groups were found in juvenile venom gels while adult gels displayed only 13 groups, revealing significant changes of *B. atrox *venom proteome during ontogenetic development.

**Figure 2:**
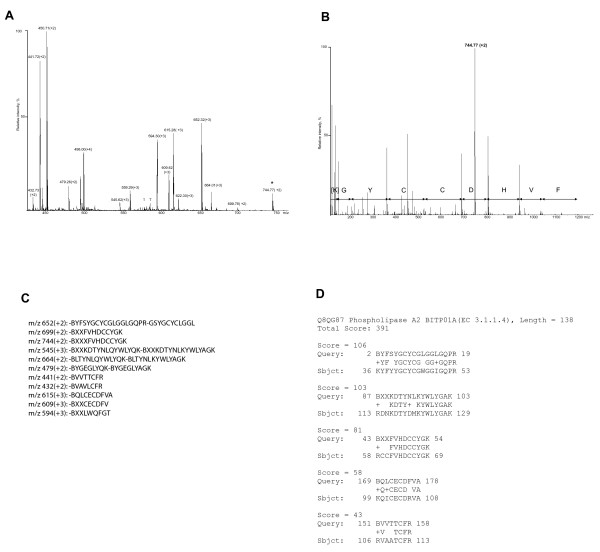
MS BLAST identification of a spot from group Y. **Panel A: **nanoES mass spectrum of the unseparated in-gel tryptic digest. Peaks of trypsin autolysis products are designated with "T". Peptide precursor ions, whose tandem mass spectra were acquired and interpreted are designated with corresponding *m/z *and charge (in parenthesis). **Panel B: **MS/MS spectrum of the precursor ion with *m/z *744.77 (designated with asterisk in panel A). *De novo *sequencing was performed by considering mass differences between the adjacent peaks in the series of fragments that belong to the ions containing C-terminal amino acid residue (y-ions), starting from high m/z region of the spectrum. The sequence shown in the panel was deduced by considering the most abundant fragment ions and was not necessarily correct; a few optional variants of interpretations based on the low abundant fragments and arriving to the typical tryptic C-terminus (K) were possible. All sequence candidates for used in a single MS BLAST search. **Panel C: **MS BLAST query that comprises all sequence proposals obtained by the interpretation of all tandem mass spectra were assembled in an arbitrary order and spaced by a minus (gap) symbol. B stands for a generic trypsin cleavage site (R or K) preceding peptide sequences and is introduced if it was possible to read out the sequence until the very N-terminus of the peptide. **Panel D: **The top confident hit of MS BLAST search. The search also reported a few homologous proteins from other species.

One spot of each group was submitted to protein sequencing by tandem mass spectrometry followed by Mascot and MS BLAST database searches (Fig. 2), which enabled either to identify the protein, or to assign it to a class of highly homologous proteins. In this way, metalloproteinases, L-amino oxidases, serine proteinases, cysteine-rich secretory proteins (CRISPs), phospholipases A2, lectins and growth factors were identified (Table 1).

Given that very few sequences of *B. atrox *proteins are available in a sequence database, the best matches of MS BLAST database searches corresponded to proteins found in venoms of other snakes, mostly of the *Bothrops *genus (Table 1). Since several snake venom proteins share high sequence similarity and peptides analyzed by MS/MS cover only a small fraction of their sequences, it was not possible to unequivocally determine the protein homologues from the sequenced species. MS/MS analysis of the spot with apparent MW of 52 kDa (group A, Fig. 1 and Table 1) that was present in similar quantities in adult, sub-adult and juvenile gels is presented here as an example. MS/MS sequencing identified it as a member of the P-III class Zn-metalloproteinase. The best matches corresponded to the jararhagin and bothropasin, high molecular mass metalloproteinases from B. jararaca, which share approximately 95% of sequence identity. One of the determined sequences (KINPFR) is present in bothropasin, but not in jararhagin and another (BMYELANIVNEIFR) shares 100% identity with jararhagin only, because there is a substitution (F→ L) in bothropasin. The remaining peptide sequences (Table 1) are present in both bothropasin and jararhagin. Therefore, the 52 kDa spot is unequivocally related to a metalloproteinase from the P-III class that is homologous, albeit is different from both bothropasin and jararhagin.

**Figure 3:**
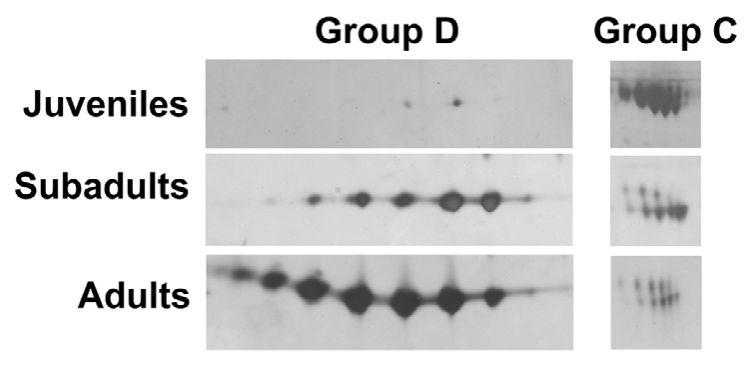
Zoomed gels showing examples of differential expression of proteins of groups D and C upon ontogenetic development.

The proteins from group C, whose molecular masses are similar to those of group A, were identified as metalloproteinases of the P-III class, albeit having higher pI values (7.1–7.5 comparing to pI 5 of group A). They share two peptide sequences (BKIPCAPEDVK and BGMVLPGTK) with the polypeptides from group A. Two other sequences (BXXVEVGEECDCGSPR and BLYCCVDSSPANK) matched bothropasin only partially and another sequence (BXXGTECQAA) occurs in metalloproteinases from other species of vipers. Differently from group A, the group C proteins are much more abundant in juveniles than adults as shown in Fig. 3.

Group D, one of the most prominent groups of spots in adult gels, comprised several isoforms of approximately 23 kDa and p*I *range between 5.3 and 6.4 (Fig 3). On the other hand, only two of these spots (in very low amounts) were detectable on silver stained gels of juvenile venom. Comparison of the three gels suggested that the concentration of 23 kDa isoforms increased during ontogenetic development (Fig. 3). N-terminal sequences of these proteins were determined by Edman degradation and the proteins were found to be homologous to bothrolysin and bothrostatin metalloproteinases (see above). MS BLAST searches with a query composed of peptide sequence proposals obtained by the *de novo *interpretation of MS/MS spectra produced as best hit a P-II class metalloproteinase from *B. insularis*. Snake venom metalloproteinases with molecular masses 23–25 kDa, which are composed by a sole metalloproteinase domain, are usually assigned to the P-I class [4,6]. Some P-I class proteins are produced by proteolytic processing of P-II metalloproteinases, which are larger and contain a desintegrin-like domain besides the metalloproteinase domain [7]. Therefore, proteolytic processing could explain the high similarity of proteins from group D with P-II class metalloproteinases. The spots from group D probably correspond to the 23 kDa polypeptide previously purified from *B. atrox *venom that constituted a single band in SDS-PAGE [2]. Further ESI-MS experiments suggested that there were at least three isoforms of this protein in the *B. atrox *venom [2]. Here, 2-DE gels were able to resolve at least seven isoforms in the venom of adults. Image analyses of the gels showed a remarkable increase in the volume of group D spots from juveniles to adults.

The group L comprised at least seven isoforms that were abundant in juvenile, less abundant in sub-adult and undetectable in adult venoms. They were homologous to berythractivase, a 78 kDa P-III class metalloproteinase found in *B. erythromelas *that is a protrombine activator that possess a high pro-coagulant activity [8].

The proteins from group M were also identified as metalloproteinases and were not detected in adults. These proteins were homologous to BOJUMET II from *B. jararacusu *as well as to berythractivase. Although the proteins from groups L and M were both homologous to berythractivase, PMF and MS/MS data indicated that they were, in fact, different proteins rather than post-translationally modified forms of the same gene.

Another group of spots (B) with molecular mass range of 55–61 kDa contained several isoforms present in similar amounts in the three different venom samples. Their sequences were homologous to apoxin I, a L-amino oxidase (LAO) from *Crotalus adamanteus*. LAO are major components of snake venoms that cause cell death by apoptosis [9].

The proteins from the groups E, N, Q and R were identified as serine proteinases. The snake venom serine proteinases possess thrombin-like activity and several of them have been isolated from bothropic venoms [4].

Proteins belonging to the class of Cysteine Rich Secretory Proteins (CRISP) were identified in group F and displayed similar expression in the three developmental stages analyzed. The CRISPs are found in epididimus and granular cells of mammals and seem to act in cell maturation of spermatozoa and cells from immune system, though the exact function of these proteins is unknown [10]. Members of CRISP family isolated from snake venoms, such as ablomin and trifilin, are responsible for blocking of smooth muscles contractions induced by depolarization. Immunological screening using anti-triflin antiserum identified CRISPs in different snake venoms, although the cross-reactivity was relatively low for the only member of the *Bothrops *(*B. jararaca*) genus tested [10].

In the low molecular mass range (12–15 kDa), several phospholipases A_2 _(PLA_2_) and C-type lectins were found. Within these groups, we identified at least four variant sequences of the same peptide stretch present in PLA_2 _(VAVLCFR, AAAVCFR, VAATCFR and VAVLYSR) indicating a high degree of polymorphism for these enzymes. Other examples of polymorphic peptide sequences were observed in groups W (more expressed in juveniles and sub-adults) and I (adult specific). MS BLAST searches produced as best hits the alpha chain of the C-type lectin bothrocetin, although the sequence of their peptides and the PMF analysis showed that they contained different proteins. C-type lectins were also identified as the major component in groups U and V. Interestingly, groups H (adults) and S (juveniles), despite having similar p*I*, molecular weight and some peptide sequences, actually contained different C-type lectins.

Proteins homologous to vascular endothelial growth factor (VEGF) from *Bothrops insularis *were detected in group X and their abundance decreased during ontogenetic development (Fig. 1). VEGFs from snake venoms are known to enhance the vascular permeability and play an important role in the initial stages of envenoming by *Bothrops*. It is assumed that snake venom VEGFs, dstimulate the distribution of the venom favouring both local and systemic actions [11].

Group Z displayed only one detectable spot that was present in juveniles and absent from adults. It was identified as a mixture of a nerve growth factor (NGF) and a C-type lectin. NGF belongs to a family of neurotrophic factors, which are endogenous soluble proteins regulating survival, growth morphological plasticity or synthesis of proteins for differentiated functions of neurons. In addition, there is increasing evidence that NGF activities are not restricted to the nervous system, but also affect non-neuronal cells, especially those of haematopoietic stem cell origin. NGF was previously described in the three main families of venomous snakes (Viperidae, Crotalidae and Elapidae). The ubiquitous presence of NGF in snake venoms suggests a toxinological importance of that protein in a sense of direct toxic action, indirect toxic action or an activity in the context of prey digestion [12].

## Discussion

The proteome composition of snake venom alters with age and therefore the developmental stage of the organism, which donated the specimen, should be taken into account. To this end, we performed a comparative analysis of *B. atrox *venom proteome in three stages of maturity. To our knowledge, this is the first proteome analysis of snake venoms associated with the ontogenetic development, although other proteomic studies on snake venom have been published previously [13,14].

In a typical proteomic routine, proteins are separated by 2-DE, visualized by silver or Coomassie blue staining [15] and protein spots *in-gel *digested with trypsin [16]. An aliquot of the digest is subjected to PMF by MALDI-TOF mass spectrometry. If no conclusive identification is achieved, tryptic peptides are then extracted from the gel matrix and sequenced by tandem mass spectrometry [17]. However, in both protein identification approaches it is required that the exact sequence of the analyzed protein is available in a database. Nevertheless, only a small number of *B. atrox *proteins are currently available in a sequence database and therefore the scope of protein identification was very limited. To address this issue, we complemented conventional protein identification methods, which are based on the exact matching of tandem mass spectra to database sequences, with sequence-similarity searches. The latter approach enabled confident identification of unknown proteins that only distantly related to known proteins from other species [18-20]. Using a combination of stringent and sequence-similarity database searches, we identified all major components of venoms and mapped out changes in the abundance of individual protein components in ontogenetically altered proteomes.

The natural polymorphism of the protein sequences, together with the absence of a complete and annotated snake genome, which could be used as a referenceid not allow the unambiguous assignment of the most related protein homologues due to the low sequence coverage. However, the group-specific assignment, based on the peptide sequences, was always reliable.

Based on peptide mass fingerprinting analysis we classified the spots of the 2-DE maps in 27 groups. Most groups and those with larger number of spots corresponded to proteinases (metallo and serine proteinases). This result agrees with the fact that venoms from *Bothrops *species are haemotoxic and promote haemorrhaging primary to extensive local swelling and necrosis [14].

The proteome maps also enabled the identification of new groups of potential ontogenetic molecular markers. For instance, we found that some groups of proteins including P-III class metalloproteinases (L, M, O and P), serine proteinases (N, Q and R) are more abundant in juvenile specimens, while metalloproteinases from class P-I (group D) are more expressed in adults.

Overall, more groups of proteins were identified in the proteome maps from juveniles, than in adults. Proteome maps from sub-adults contained spots from both juveniles and adults, along with a few stage-specific spots. Previous work suggested that the *B. atrox *venoms from young individuals trigger more potent biological effects than adult venoms [2,5]. The decrease in hemorrhagic activity in *B. atrox *venoms during ontogenetic development may be explained by the lower levels of Zn-metalloproteinases of P-III class in adults comparing to juveniles. On the other hand, the higher concentration of berythractivase, a pro-thrombin activator, may reflect the higher coagulant activity of juvenile venoms. The effect of VEGFs, NGFs and CRISPs, all more expressed in young specimens, certainly also contribute to the higher pharmacological activities of the juvenile venom.

The diversity in the protein composition and biological activity of snake venom during growth may be related to adaptation by evolutionary processes to the type and size of the prey [21-24]. Young *Bothrops *snakes preferentially eat amphibians, lizards, birds, and shift to mammals when they become adults [25]. Therefore, the qualitative and quantitative changes in the *B. atrox *venom proteome are most likely related to the survival of the snake by prey adaptation.

## Conclusion

We have established proteome maps for the venom of *B. atrox *in three different developmental stages, i.e. juvenile, sub-adult and adult. Analysis of the proteome maps confirmed that *B. atrox *venom proteome alters significantly with aging of the animal. Moreover, we have verified the existence of stage specific and differentially expressed polypeptides that may be responsible for the diverse activities of the venom in each developmental stage analyzed.

The proteome of any biological sample is expected to dynamically change in response to external stimuli. The changes in the *B. atrox *venom proteome reported here reinforce the need to intensify the studies on organisms in different stages of maturation. This procedure may lead to the discovery of a wider group of molecules of biotechnological and medical interest, as well as to better understanding of important biological traits of the organism during its ontogenetic development.

Finally, intra-species variations in the composition of snake venom during ontogenetic development prompts further studies of the relationship between symptomatology of snake bite accidents in humans with the venom composition as well as the use of venoms from individual specimen of various ages for the production of antivenoms.

## Methods

### Snake venoms

Venoms were obtained from wild *Bothrops atrox *specimens with no known litter relationships, captured in Manaus region (Amazonas State, Brazil), and maintained in the Herpetarium of the Gerência de Animais Peçonhentos-IMT-AM, Manaus. Classification of *B. atrox *snake specimens was done based on the total lenght of wild specimens according to Martins and Oliveira (1998) [26]. Therefore, three size classes were defined: juveniles (≤ 40 cm), sub-adults (> 40 – 70 cm), and adults (> 70 cm). Twenty days after the snakes were captured and before their first feeding in captivity, the venoms were manually milked by massaging the venom glands of specimens longer that 50 cm, and with the aid of a Pasteur pipette on snakes smaller than 50 cm. A single extraction was performed for each specimen. Three types of pooled venom samples were prepared, juvenile, comprising venoms of eight specimens, sub-adult, corresponding to twenty five specimens and adult, a mixture of venoms of twelve specimens. The pooled venom samples were centrifuged, filtered, lyophilized, weighted and stored at -20°C.

### Two-dimensional gel electrophoresis

Freeze dried pooled venom samples (100 μg for silver stained gels and 300 μg for Coomassie blue stained gels) were solubilised in 370 μL of 2-DE sample buffer (7M urea, 2M thiourea, 1% DTT, 2% Triton X-100, 0.5% Pharmalyte 3–10) containing proteinase inhibitors (100 μM PMSF, 1 μM pepstatin A and 5 mM EDTA) and applied to 18 cm IPG gel strips (GE Healthcare, Upsala, Sweden) containing linear or non-linear 3–10 pH gradient by in-gel sample rehydration [27]. After 12 h of rehydration in the sample solution, isoelectric focusing (IEF) was carried out at 20°C using an IPGphor unit (GE Healthcare) in three successive steps: 500 Vh, 1000 Vh and 32000 Vh. For reduction and alkylation the IPG gel strips were soaked for 30 min in a solution containing 50 mM Tris pH 8.8, 6 M urea, 30% glycerol, 2% SDS and 125 mM DTT and for an additional 30 min in the same buffer containing 125 mM iodoacetamide and bromophenol blue instead of DTT. SDS-PAGE was performed on 10–20% T polyacrylamide gradient gels on a Protean II system (Bio Rad, Hercules, CA, USA) connected to a Multitemp II cooling bath (GE Healthcare). Electrophoresis was carried out at constant current (25 mAmp *per gel*) at 20°C until the dye front reached the lower end of the gel.

2-DE gels containing 100 μg of venom were silver stained [28] and submitted to image analysis. Alternatively, 2-DE gels containing 300 μg of venom were stained with 0.1% (w/v) Coomassie Blue in 40% (v/v) methanol, 10% (v/v) acetic acid and distained with the same solution without Coomassie Blue.

### Image analysis

Silver stained gels were scanned with a SHARP JX-330 scanner (Tokyo, Japan) at 300 dpi resolution and the *tiff *images generated were analyzed with Image Master 2D Elite software (GE Healthcare). Spot detection and spot matching were performed in automated mode. Spot volumes were acquired without background subtraction and normalized using the total intensity of the detected spots.

### Protein digestion

Coomassie stained spots were excised from the preparative gel and *in-gel *digested with trypsin as described in [15].

### Peptide mass fingerprinting

Protein digests were first subjected to peptide mass fingerprinting by matrix-assisted laser desorption/ionization on a Bruker Reflex IV time-of-flight (MALDI-TOF) mass spectrometer equipped with Scout 384 ion source. Probes were prepared by dried-droplet method as described previously [29]. Briefly, 1 μL aliquot of the digest was mixed on the surface of AnchorChip™ 384/600 targets (Bruker Daltonics, Germany) with a saturated solution of matrix (α-cyano-4-hydroxycinnamic acid) in 1: 2 (v/v) solution of 2.5% aqueous TFA: acetonitrile. The mixture was allowed to dry at room temperature and the entire target was washed with 5 % formic acid.

Spots whose peptide mass fingerprints were similar, i.e. shared more than 75% of peaks with the relative intensity of more than 20 % within the mass accuracy of +/- 100 ppm, after removal of peaks of trypsin autolysis products and known keratin contaminants, were grouped as shown in Fig 1.

### Sequencing by nanoelectrospray tandem mass spectrometry

If peptide mass fingerprinting did not identify the protein, peptides were extracted from the gel pieces with 5 % formic acid and acetonitrile and the extracts were pooled together and dried down in a vacuum centrifuge. The digests were taken up in 5 % formic acid and analyzed by nanoelectrospray tandem mass spectrometry on a hybrid quadrupole time-of-flight instrument QSTAR Pulsar *i *(MDS Sciex, Canada) as previously described [30,31].

### Database searching

Peptide mass fingerprints were used for database searching using Mascot software (Matrix Science Ltd, UK) against the MSDB database (updated May 15, 2005; containing 2011425 protein sequence entries) downloaded from NCBI. Mass tolerance was set to 100 ppm, spectra were calibrated externally and no restrictions were imposed on protein molecular mass or phylogenetic lineage. Uninterpreted tandem mass spectra were first searched by Mascot against the above database to identify proteins with tryptic peptides identical to database entries. Precursor mass tolerance was set at 0.1 Da and fragment ion mass tolerance at 0.05 Da. Hits were considered significant if their protein score exceeded the threshold score calculated by Mascot software assuming *p *< 0.05. Matched MS/MS spectra were further manually inspected considering the correlation of *y-*, *b- *and *a- *fragment ions [32] to corresponding *m/z *calculated from the peptide sequences. If still no identification was achieved, tandem mass spectra were interpreted *de novo *using BioAnalyst QS software as previously described [31]. Multiple sequence candidates were allowed per each interpreted tandem mass spectrum and partial peptide sequences were included into the search string. All candidate sequences were merged into a single search string and MS BLAST searches were performed against a non-redundant protein database (nrdb) at  or  under default settings. Parsing script operating at the MS BLAST web site was applied to identify and colour code statistically confident hits according to MS BLAST scoring scheme [20]. According to the selected MS BLAST threshold scores of statistical confidence, the expected rate of false positive identification was lower than 1 %.

## Abbreviations

2-DE: two-dimensional gel electrophoresis, IPG: immobilized pH gradient, CRISP: Cysteine Rich Secretory Proteins, PLA_2_: phospholipase A_2,_VEGF: vascular endothelial growth factor, NGF: nerve growth factor, IEF: isoelectric focusing, DTT: dithiotreitol, PMSF: phenylmethyl sulfonyl fluoride, EDTA: ethylenediaminetetraacetic acid.

## Competing interests

The author(s) declare that they have no competing interests.

## Authors' contributions

RAPG- Participated in 2D-PAGE, protein blotting, data analysis and writing of the manuscript.

Anna S – Peptide mass fingerprinting, MS/MS analysis and database searching.

Andrej S- Database searching and data analysis. Participated in the writing of the manuscript

JLLP- Animal collection, venom sample preparation and general discussion of results

J. P- Optimization of 2D-PAGE conditions and computational image analysis

MVS- Participated in the design of the study, coordination and writing.

CAOR- Experimental design, data analysis and coordination. Preparation of the final version of the manuscript.

All authors read and approved the final manuscript.
